# The implementation of reusable drapes and gowns in operating theatres: A mixed-methods analysis of data from 5230 peri-operative professionals in 134 countries

**DOI:** 10.1186/s43058-025-00732-x

**Published:** 2025-06-02

**Authors:** Virginia Ledda, Aneel Bhangu, James Glasbey, Elizabeth Li, Antje Lindenmeyer, Sivesh Kamarajah, Dion Morton, Maria Picciochi, Dmitri Nepogodiev, Laura Kudrna

**Affiliations:** 1 NIHR Programme Grant for Environmentally Sustainable Surgery, Institute of Applied Health Research, University of Birmingham, Birmingham, UK; 2 Institute of Applied Health Research, University of Birmingham, Birmingham, UK

## Abstract

**Background:**

Reuse of sterile textiles could potentially reduce the carbon footprint of operating theatres. The aim of this mixed-methods study is to gain a deeper understanding of the implementation of reusable drapes and gowns across different contexts through applying the Consolidated Framework for Implementation Research (CFIR).

**Methods:**

A mixed-methods analysis was performed of cross-sectional data from a survey distributed by snowball sampling across a global research network. A five-point Likert scale assessed perspectives on safety and feasibility of implementation of reusable textiles. An open-ended question asked about the implementation of reusables. Inductive and deductive coding was used, informed by the CFIR.

**Results:**

A total of 5734 responses were collected, of which 5230 were eligible for inclusion. Most respondents believed that the intervention was unlikely or very unlikely to affect safety (3266/5230, 62.4%) or have detrimental consequences on environment or patient care (2990/5230, 57.2%), and that its introduction was likely or very likely to be successful (2888/5230, 55.2%). From a total of 1514 free-text entries, nine unique implementation factors were identified. Financial constraints were important across all settings. Whilst sterilisation-related issues were commonly reported in low and middle-income countries, institutional challenges were prominent in high-income countries. Mapping these factors to CFIR, the ‘setting’ of the intervention was the most relevant for effective implementation, along with the ‘individuals’ within the setting, rather than the intervention itself or the implementation process. A strategy identification tool and programme theory were developed, providing a starting point for institutions considering implementation of reusables and basis for future research.

**Conclusion:**

Implementation of this carbon-reducing intervention varied across resource settings. Recognising the unique implementation context, and developing tailored strategies, could aid implementation of this innovation through both research and wider scale rollout.

**Supplementary Information:**

The online version contains supplementary material available at 10.1186/s43058-025-00732-x.

Contribution to the literature

• Given the need for health systems worldwide to implement ‘green’ practices to reach net-zero, it is vital to gain an understanding on how these can be effectively introduced, implemented and sustained.
• The research provides a comprehensive account of the factors affecting use of reusable textiles in hospitals globally, making a novel contribution to bridging environmentally sustainable surgery and implementation research
• The findings show that barriers change according to different contexts and provide important knowledge to plan further research and implement ‘green’ practices in hospitals worldwide to tackle climate change.

## Background

Operating theatres are the most energy-intensive part of hospitals, requiring constant heating, ventilation and air conditioning. They rely heavily on single-use items, generating significant waste, and releasing anaesthetic gases. Decarbonising operating theatres is a complex but essential step for health systems aiming to achieve net-zero targets. Switching to reusable textiles has been recommended as a measure to reduce the environmental impact of operating theatres [1, 2]. Currently, a mixed practice of reusable and disposable drapes and gowns is adopted across high and lower and middle income countries [3]. The World Health organisation (WHO) does not provide guidelines on the use of single-use versus reusable drapes and gowns, due to the lack of robust evidence comparing their effect on SSI rates [4]. Switching to reusable textiles could save a 1,000-bed hospital £100,000 in procurement and clinical waste costs and 20 tonnes of waste per year. Reusable textiles have also been shown to be more comfortable than their disposable counterparts [5, 6]. Life-cycle assessments have shown that reusable textiles have reduced environmental impact, compared to their disposable counterparts [6, 7]. The use of reusable textiles, in comparison to their disposable counterparts, was found to be associated with a reduction in greenhouse gases emissions by 66%, and solid waste generation by 84% per 1,000 uses [7]. NHS England modelling shows that switching to reusable gowns could save 100 tonnes of CO_2_e annually for a 1,000 bed hospital. This equals the amount of CO_2_e emitted by the electricity consumption of 25 households for a year or by driving a car for 500,000 km [5]. These studies are set in high-income countries and as such they fail to address the variation in supply chain and sterilisation systems across different resource settings. There is a paucity of high-quality evidence on the environmental impact of drapes ad gowns in low-middle income countries. Further evidence is required to justify and support changes in hospital practices worldwide.

To deliver high-quality, robust results that drive changes in practice, research must ensure adherence to interventions. Achieving this, alongside fostering long-term implementation, requires a thorough understanding of the challenges and opportunities associated with implementing reusable drapes and gowns, as well as how these vary across different contexts. The application of implementation science frameworks in surgery is recognised as an important but overlooked opportunity for improving quality and safety in surgical care [8]. This research considers how the application of an implementation science lens and investigation of context can add value to this field and extend the focus on quality and safety to the environmental sustainability of surgical practices. By adopting a sensitive contextual lens, it also provides a structured and comprehensive approach to assessing barriers to implementation, helping to identify the contextual factors that will determine the success (or failure) of future implementation projects.

This mixed-methods study aims to gain a deeper understanding of the perceived barriers to the introducing reusable drapes and gowns across different contexts, and to map these barriers to the existing Consolidated Framework for Implementation Research (CFIR). The study also proposes strategies to overcome these barriers and contributes to the development of a programme theory for future research and implementation studies in sustainable surgery.

## Methods

A global consultation survey of peri-operative professionals was previously undertaken to understand what interventions could be introduced globally to reduce the environmental impact of surgery, and what their barriers across different settings would be. The survey consisted of two rounds. The first round aimed to co-prioritise and rank a longlist of interventions, previously identified through systematic review, to identify the most feasible, acceptable, and safest. Results identified a variety of interventions as possible targets including reducing desflurane use, switching to reusable textiles, and optimising waste management. The results of the first round of the global survey have previously been published in a separate peer-reviewed research paper [9].

The second round of the global survey focussed on the perceived barriers to each individual intervention and assessed views on safety, possible unintended consequences, and likelihood of success. This study focusses on analysing unpublished responses to the second round of the survey, relating to the intervention ‘Introduce reusable surgical drapes and gowns’ as a strategy to reduce the environmental impact of operating theatres.

### Theoretical framework

The reporting of this study follows the Standards for Reporting Qualitative Research (SRQR) by the Equator network (Supplementary Material 1) [10]. Given the subjective nature of the qualitative component of this study, the characteristics of the researchers involved in the study are described in Supplementary Material 2. This ensures transparency, by considering potential bias and assumptions of the researchers based on their background, field of study and previous research experience [11].

The study design was a cross-sectional mixed-methods survey. The survey collected both quantitative and qualitative data. It was informed by the Creswell principle of Explanatory Sequential Design, with the purpose for qualitative data to further characterise the initial quantitative responses [12]. The free-text responses within the study were qualitatively analysed and used to identify the barriers to the implementation of reusable drapes and gowns. This analysis was embedded within a constructivist paradigm, as the responses collected provided insights into the participants’ experiences, allowing the researchers to build knowledge around the topic [13].

A variety of implementation science frameworks exist that could be utilised to understand the implementation of reusable drapes and gowns in operating theatres globally, including the Reach, Effectiveness, Adoption, Implementation, Maintenance (RE-AIM) and the Normalisation Process Theory (NPT) [14, 15]. Tools exist to support the selection of the most appropriate framework to match the study objectives, such as the Theory, Model and Framework Comparison and Selection Tool (T-CaST). However, the Consolidated Framework for Implementation Research (CFIR) was selected for this study because of its rich consideration of contextual factors and recent integration with a prominent individual-level model of behaviour change, the behaviour change wheel (BCW) [16]. The BCW was developed through systematic review of existing approaches and frameworks with expert consensus, and, as such, it has important value in thinking about why individual actors within inner and outer contexts and systems behave [17].

In brief, the CFIR facilitates the structured assessment of deterministic barriers and facilitators and aids the design and delivery of interventions and their adaptations to different contexts. The framework consists of five broad domains, which describe the different contexts to consider when implementing an intervention at organisational level. These include the intervention itself (Innovation), the setting where the intervention is implemented (Inner setting), the individuals within the setting (Individuals), the surrounding environment (Outer setting) and the strategies through which the intervention is implemented (Implementation process). Within these, thirty-nine constructs are included, representing elements affecting implementation in different contexts [18].

The updated framework also includes, within the Individuals domain, constructs from the inner layer of the Behavioural Change Wheel (BCW). The wheel aids the selection and design of interventions chosen to lead to changes in individuals’ behaviour. CFIR draws on the innermost part of the wheel, Capabilities, Opportunities and Motivation as three factors influencing the ability of an individual to change behaviour. Capabilities indicate the knowledge and skills needed to engage in a behaviour, Opportunities are the external factors that allow its execution. Motivation is the conscious and unconscious mental processes guiding an individual towards the behaviour [17]. Therefore, CFIR was selected to support this study as it focuses on contextual factors affecting implementation at multiple levels. This was found particularly relevant in a study looking at the implementation of reusable drapes and gowns in different settings at a global level.

### Data collection and processing

Participants for the survey were sampled from across the National Institute for Health and Care Research (NIHR) Global Health Unit on Global Surgery network following the principles of snowball sampling. Participants were included if they worked as frontline peri-operative professionals, to gain insight from experiences of staff within the operating theatres. Professionals eligible to be involved in the study included anaesthetic doctors and nurses, surgeons from any surgical specialty, operating department practitioners, physician assistants and theatre staff including theatre nurses, managers, and engineers. Respondents from any country and any type of hospital setting were invited to participate to the survey.

The survey consisted in a stakeholder engagement, with fully anonymized expert opinions collected from our network of research collaborators and co-authors, and no patient involvement. According to INVOLVE guidance, ethical approval is not required for research activities involving patients and the public when they have active involvement, providing opinions and specialist knowledge or advice [19].

The survey was completed between the 30th of January and the 5th of February 2022. This included five-point Likert scale close-ended questions, aiming to summarise views on reusable textiles. An open qualitative item to enquire about perceived potential barriers to the intervention was also included. Data on participant characteristics was also collected, including their country of origin and job role (Table S1).

The data was collected and stored online through a secure server running the Research Electronic Data Capture (REDCap) web application. The service was managed by the Global Surgery REDCap system hosted at the University of Birmingham, United Kingdom, and its security was governed by the policies of the University of Birmingham.

Following completion of the data collection process, the data was exported on Microsoft Excel. The data was checked for completeness and non-eligible, duplicate, and blank responses were excluded from the analysis.

### Data analysis

Quantitative analysis was conducted on data from five-point Likert scale questions included in the survey. Responses were analysed and descriptive percentages provided, mapped to income groups as defined by the World Bank classification (high, upper middle, lower middle and lower income countries) [20].

The inductive coding of the data was informed by an adapted version of the Spradley domain analysis methodology, which focusses on identifying key areas of content within a common topic, in this case barriers, and the relationship among these areas [21].

VL initially performed inductive open coding of the free-text responses, assigning codes to phrases that reflected specific concepts or ideas. These initial codes were then iteratively grouped into higher-level codes representing perceived barriers to the proposed intervention. The higher-level codes, sub-codes and their descriptions were compiled into a coding frame, which was subsequently used to complete the coding of the full set of free-text responses. Simultaneous coding was performed, as segments from one free-text response could be assigned multiple codes, reflecting the variety of ideas present within one response [22]. The coding frame was iteratively reviewed, with sub-codes collapsed and collated as appropriate, with researcher triangulation including VL, LK and DN. A final coding frame contained higher level codes, representing the main perceived barriers to the introduction of reusables drapes and gowns, and sub-codes, providing nuanced detail about each barrier. Descriptive percentages on the frequency of coding across different income groups were supplied, based on the first code assigned to each free-text response (dominant code) [22].

The codes and sub-codes within the coding frame were then deductively mapped to the domains of the updated Consolidated Framework for Implementation Research (CFIR) [18].

Mapping perceived barriers reported by peri-operative professionals to CFIR allowed to gain a deeper understanding of the organisational, institutional, and individual level factors that can be addressed to influence the uptake of reusable drapes and gowns.

VL, the first coder, initially mapped the codes and sub-codes to the CFIR framework. A research triangulation meeting was subsequently held with VL, LK and DN resulting in full consensus.

Between the inductive and deducting coding phases, 10% random sample of responses were double coded (VL, LK), and inter-rater reliability was measured through calculation of Cohen’s kappa to assess the consistency of coding between the raters [23]. This was based on the higher-level, dominant code.

### Strategy identification tool

A strategy identification tool was developed to aid in the identification of implementation strategies for institutions wishing to introduce reusable textiles. The practical actions included were identified from the literature on implementation strategies and the CFIR Interview Guide Tool [24, 25]. The Interview Guide Tool was built to support gathering of qualitative data to evaluate the implementation of an intervention according to the CFIR framework [25]. The practical actions were initially adapted by VL, and further refined through consultation with AB, DN and LK.

### Programme theory

The knowledge provided by scoping of the existing literature, as well as the results of the quantitative and qualitative analyses reported, were integrated and allowed the development of a programme theory on the use of reusable drapes and gowns. A template was developed drawing on existing National Institute for Health Care Global Health Research Centres guidance for the development of a programme theory of change and the Medical Research Guidance for developing and evaluating complex interventions [26, 27]. The programme theory was reviewed in light of realistic approaches to programme evaluation [28]. The activities within the programme theory were targeted to address the barriers emerged from the qualitative analysis.

## Results

A total of 5734 responses to the survey were collected. Of these, 45 were excluded as the respondent was ineligible to participate, 188 were excluded as all data were missing, and 271 were excluded as duplicates. Of the remaining 5230 responses, 1563 provided answers to the open-ended questions, but 49 were incomplete sentences or their content did not relate to reusable drapes and gowns. Therefore, 5230 responses were included in the quantitative analysis and 1514 responses were included in the qualitative analysis (Figure S1).

Overall, over half of the respondents were from high-income countries (HICs, *n* = 2995, 57.2%), with less than half from low- and middle- income countries (*n* = 2235, 42.7%). Most of the respondents were surgeons (*n* = 4327, 82.7%), with other respondents including anaesthetists and anaesthetic nurses, theatre staff and physician associates (Table 1).

**Table 1:** Survey respondents’ demographics

Survey Respondents	*n* = 5230	
**World Bank Country Income Classification**		
High income countries	2995	57.3%
Low- and middle-income countries	2235	42.7%
Upper middle-income countries	879	16.8%
Lower middle-income countries	1069	20.4%
Low-income countries	287	5.5%
**Job roles**		
Anaesthetist	481	9.2%
Anaesthetic nurse	10	0.2%
Operating department practitioner	93	1.8%
Physician assistant	232	4.4%
Surgeon	4327	82.7%
Theatre manager/ stores/ engineer	35	0.7%
Theatre nurse	51	1.0%
* Missing*	1	0.0%

### Quantitative analysis

A majority of respondents believed that the intervention was either unlikely or very unlikely to affect safety of patients (3266/5230, 62.4%). Overall, 1165 out of 5230 respondents (22.3%) thought this likely or very likely, while in low-income countries (LICs) 117 out of 287 (40.8%) thought this likely or very likely.

A majority of respondents also believed that the introduction of reusable drapes and gowns was unlikely or very unlikely to have other unintended detrimental consequences on the environment or on patient care (2990/5230, 57.2%, Table S2). Overall, 1236 out of 5230 respondents (23.6%) thought this likely or very likely, whereas in LICs 110 out of 287 respondents (38.3%) thought this was likely or very likely.

Over half of the respondents thought introduction of reusable drapes and gowns was likely or very likely to be successful (2888/5230, 55.2%). This result was consistent across upper- middle (UMICs), lower middle- (LMICs) and LICs (Table S1). However, opinions in high income countries were more ambivalent with 23.0% of respondents reporting the introduction was unlikely to be successful (690/2995, Table S2).

### Qualitative analysis phase 1- Inductive coding

Of the 1514 answers to the open-ended questions, 263 (17.4%) reported no barriers or stated that the reusable drapes and gowns had already successfully been adopted. Through inductive coding of the remaining 1251 responses, a final coding frame containing nine higher-level codes and forty sub-codes was developed, shown in Table 2 alongside example quotations from survey free-text responses and in Table S3 with descriptions of each subcode.

**Table 2:** Coding frame with example quotations from free-text responses

Code	Sub-code	Example quotations
**Evidence**	**Further evidence required**	‘The evidence of the safety and efficacy of such materials must be undebatable’ ‘The opinion expressed that more data from the studies are needed regarding whether their use is beneficial in reducing the SSI rate’
	**Lack of evidence- Asepsis**	
	**Lack of evidence- Cost benefit**	
	**Lack of evidence- Environmental benefit**	
**Finance**	**Cost**	‘Cost of maintenance and sterilisation is high, no financial benefit.’ ‘Initial investment costs to purchase gowns, and costs of cleaning’ ‘There are costs associated with laundry and the outlay to purchase reusable drapes / gowns is often more expensive.’
	**Cost of initial investment**	
	**Cost of sterilisation**	
	**Cost of workforce**	
	**Cost-other**	
**Institution**	**Institutional barriers**	‘Would require a whole new way of working—organisations very resistant to change.’ ‘This intervention would require significant planning and change of current workflows.’ ‘We had reusable gowns and drapes in past but the policy of the hospital is to abandon them to reduce cleaning and sterilization costs… now we have one-use gowns and drapes.’ ‘Our hospital infections-commission decided one-use surgical gowns and drapes better than reusable ‘
	**Bureaucratic barriers**	
	**Environmental sustainability not prioritised**	
	**Resistance from stakeholders**	
	**Institutional guideline or policy**^**1**^	
	**Previous switch to disposable**	
	**Change in workflow**	
	**Lack of motivation**	
	**Single- use culture**	
**Intervention**	**Quality of drapes and gowns**	‘The reusable gowns or drapes are made of fabric (at least in my country), so they are not appropriately waterproof therefore (…) they are not a good option.’ ‘A retrograde step—re-useable gowns. Drapes that were never properly secured to the patient’
	**Practicality and Comfort**	
	**Negative perception of intervention**	
	**Permeability of reusables**	
**Procurement**	**Procurement**	‘Long lasting contracts for single use drapes and gloves’ ‘Theatre users worried about stock of reusable gowns/drapes in unexpectedly busy periods’
	**Shortage in supply of reusables**^**2**^	
**Resources**	**Additional resources required**	‘Energy and resources needed for sterilizing re-used gowns and drapes, not enough personnel to do so 24/7 ‘ ‘Additional workload required for specialised laundry sterilisation’
	**Additional workforce required**	
	**Additional workload with reusables**	
	**Additional time required**	
**Safety**	**Concerns about safety**	‘Concerns about infection transfer and adequate cleaning of these previously used items’ ‘still major concern around prions and need to use disposable equipment ‘
	**Concerns about infection/ contamination**	
	**Disposable to be used for high-risk cases**	
**Sterilisation**	**Sterilisation process**^**3**^	‘1- no enough sterilisation methods. 2- likely to be used incorrectly or not wisely.’ ‘Ensuring a sterilisation process to allow gowns to be reusable needs to meet certain standards ‘ ‘Sterilisation issue due to lack of clean water and poor cut off’
	**Sterilisation equipment**	
	**Concerns about inadequate sterilisation**	
	**Sterilisation-other**	
**Other**	**Education and training**	‘Hard to educate senior doctors’ ‘We sometimes use reusable drapes but I always prefer disposable ones’ ‘Increased washing associated with negative environmental effects of laundry services’
	**Environmental concern**	
	**External barriers**	
	**Individual preference**	
	**Other**	

1: includes ‘Approval from infection control team, 2: includes Sterilisation causing delays in supply, 3: includes Lack of basic utilities

Overall, the most frequently coded barriers to implementing reusable drapes and gowns were financial constraints (249/1251, 19.9%), sterilisation-related issues (235/1251, 18.8%) and institutional-level factors (214/1251, 17.1%). Other higher-level codes included safety concerns (123/1251, 9.8%), the intervention itself (77/1251, 6.2%), procurement of drapes and gowns (73/1251, 5.8%), and lack of resources or evidence (62/1251, 5.0%) (Table 3). Within the ‘other’ code, factors included education and training, concerns about the environmental effects of using reusable textiles, surgeons’ individual preferences and barriers external to the institution.

**Table 3:** Frequency of coding of barriers, presented by country income groups

	**HIC** **(*n* = 840)**	**UMIC** **(*n* = 145)**	**LMIC** **(*n* = 226)**	**LIC** **(*n* = 40)**	**Total** **(*n* = 1251)**
Evidence	49 (5.8%)	6 (4.1%)	7 (3.1%)	0 (0.0%)	**62 (5.0%)**
Finance	166 (19.8%)	29 (20.0%)	42 (18.6%)	12 (30.0%)	**249 (19.9%)**
Institution	169 (20.1%)	23 (15.9%)	20 (8.8%)	2 (5.0%)	**214 (17.1%)**
Intervention	53 (6.3%)	5 (3.4%)	15 (6.6%)	4 (10.0%)	**77 (6.2%)**
Procurement	46 (5.5%)	11 (7.6%)	12 (5.3%)	4 (10.0%)	**73 (5.8%)**
Resources	45 (5.4%)	5 (3.4%)	10 (4.4%)	2 (5.0%)	**62 (5.0%)**
Safety	80 (9.5%)	19 (13.1%)	21 (9.3%)	3 (7.5%)	**123 (9.8%)**
Sterilisation	121 (14.4%)	24 (16.6%)	79 (35.0%)	11 (27.5%)	**235 (18.8%)**
Other	111 (13.2%)	23 (15.9%)	20 (8.8%)	2 (5.0%)	**156 (12.5%)**

*HIC* high-income countries, *UMIC* Upper middle-income countries, *LMIC* lower middle-income countries, *LIC* low-income countries

While financial constraints were the commonest or second commonest barrier across all country income groups, institutional-level factors emerged as the most common barrier in HICs (169/840, 20.1%). Sterilisation-related issues were identified as the main barrier in lower income countries, mentioned by 79 out of 226 respondents (35.0%) in LMICs and 11 out of 40 respondents (27.5%) in LICs (Table 3).

In the subgroup of respondents who stated that the intervention was ‘Unlikely’ or ‘Very Unlikely’ to be successfully implemented in their institution (*n* = 488, Table S4), results resembled those of the overall analysis. Financial constraints (117/488, 24.0%) were identified as the commonest barrier, followed by institutional-level factors, (97/488, 19.9%) and sterilisation-related issues (77/488, 15.8%). In the subgroup of orthopaedic surgery respondents, safety did not emerge as the most common barrier (17/131, 13.0%), with financial constraint being the most coded barrier (26/131, 19.8%, Table S5).

Based on double-coding of a 10% random sample of responses, Cohen’s kappa was calculated as 0.81, indicating a strong level of inter-rater agreement [23].

### Qualitative analysis phase 2- Deductive coding and framework mapping

**Fig. 1:**
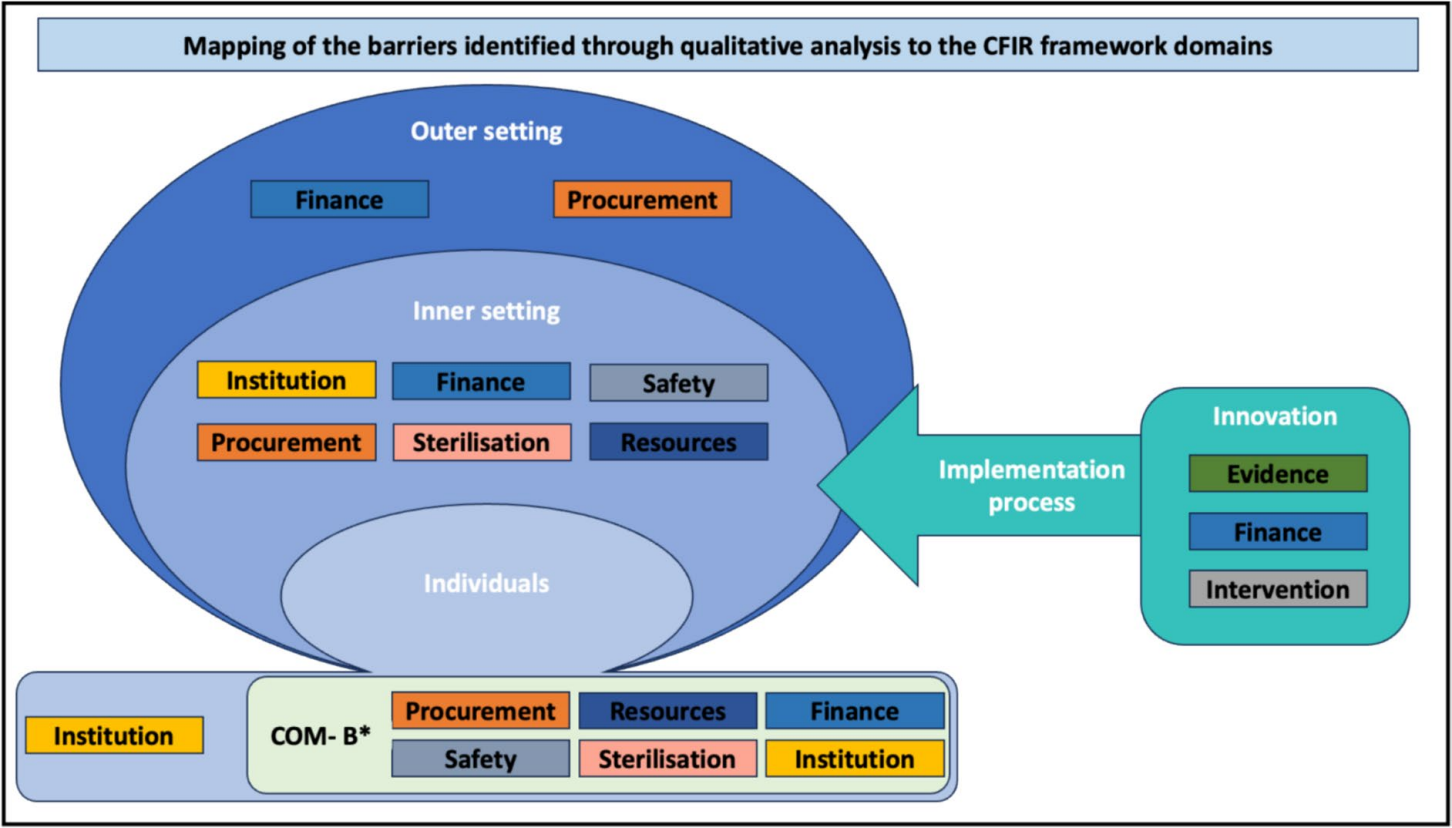
Mapping of the barrier codes to the CFIR framework. ‘Other’ barriers mapped to ‘Outer setting’, ‘Inner setting’ and ‘Innovation’. Full mapping including CFIR constructs and subconstructs available in Figure S2. CFIR: Consolidated Framework for Implementation Research. *COM-B: Capability Opportunity Motivation- Behaviour. Barriers contained in this box are mapped to the COM-B constructs within the Individuals domain of the CFIR framework

A summary of the mapping to CFIR is displayed in Fig. 1. Overall, most of the barriers mapped to the Inner setting and Individual domains of the CFIR framework. Within the Inner setting domain, most barriers mapped to the Work Infrastructure (the organisation of tasks and responsibility within teams) and Compatibility (how the innovation fits within workflow and processes of the institution) constructs (Figure S2).

Within the Individuals domain, most of the barriers identified in the study mapped to the Opportunity construct described in the COM-B model, with the barriers of finance, procurement, sterilisation, and resources all mapping to this construct. The Innovation and Outer Setting domains were found to be somewhat less influential to the implementation of the intervention. None of the barriers were mapped to the Implementation process domain which is consistent with the framing of the question on barriers in the survey questionnaire (Fig. 1, S2). A single barrier may manifest differently in different settings. Procurement for example, can be identified in the Outer setting as the ‘lack of affordable, reliable suppliers’, while in the Inner Setting as the delay in supply caused by sterilisation. Within the Individuals domain, the Procurement barriers identified those individuals in charge of procurement and their resistance to change suppliers. These nuances can be further explored by examining Table 2, which contains quotes from the respondents, and Figure S2.

### Strategy identification tool

**Fig. 2:**
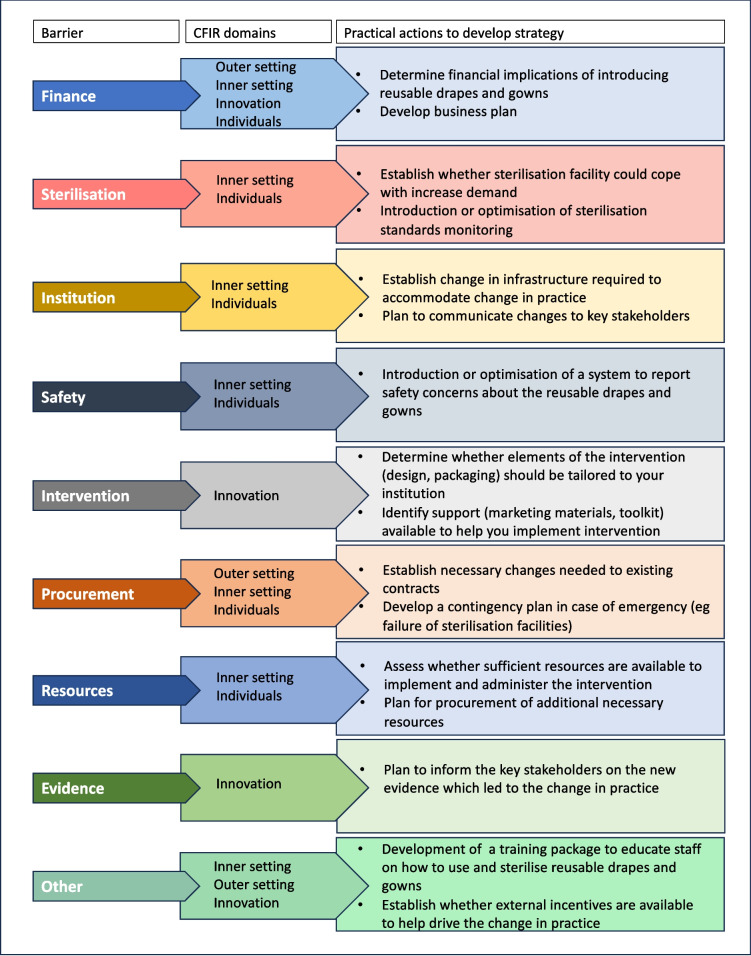
Strategy identification tool. CFIR: Consolidated Framework for Implementation Research

Based on the framework mapping, a strategy identification tool was developed to assist institutions considering implementation of reusable drapes and gowns. The tool (Fig. 2) incorporates the barriers identified in the study, the CFIR domains to which these barriers mapped, and practical actions to support institutions in the identifying and developing the most suitable implementation strategies to overcome each barrier. The tool serves as a starting point, providing a roadmap to help stakeholders tailor strategies for the effective implementation of reusable drapes and gowns at their institution.

### Programme theory

The programme theory is shown in Figure S3. The contextual factors identified from the survey data and a review of the existing literature have been summarised, focusing on the geographical location of the hospital, its characteristics, and the current practices of use of drapes and gowns. The inputs include the resources required to implement the use of reusables and the workforce and individuals involved, which are categorised according to the CFIR domains (Inner setting and the Individuals within this, the Outer setting and Innovation). The arrows in the figure illustrate some potential relationships; however, other relationships may exist that are not depicted, which could lead to different outcomes or unintended consequences. For example, within a small hospital with mixed practice (using both reusable and disposable drapes and gowns), effective communication and collaboration among frontline teams, sterilisation facility staff and infection control teams could improve sterilisation standards and enhance efficiency through staff education and training. However, a lack of involvement of the infection control team or inadequate staffing could result in insufficient sterilisation capacity, delays in operation, or suboptimal sterilisation, potentially increasing the risk of surgical site infections.

## Discussion

### Key findings

The key finding of this study was that financial constraints, sterilisation-related issues, and institutional-level factors are the main perceived barriers to implementation of reusable textiles.

Other findings reveal that more than half of the respondents believe reusable drapes and gowns are unlikely to affect patient safety or have detrimental consequences on the environment or patient care, and that they could be successfully introduced within their hospital. Mapping of the barriers identified to the CFIR framework domains demonstrated that the majority of the barriers are related to the setting where the intervention is being implemented, and to the individuals working within that setting. Notable differences in barriers were identified across respondents from different income groups, with Financial constraints being recognised as a main barrier in LICs, Institutional factors being particularly relevant in HICs and Sterilisation-related issues in LMICs and LICs.

### Current evidence

The WHO does not make global recommendations on the use of reusable or disposable drapes and gowns due to lack of robust clinical data on their impact on SSIs across diverse resource settings [23] The International Organization for Standardization (ISO) sets requirements for the undertaking and monitoring of sterilisation processes, ensuring the sterility of textiles until use [29]. While organisations such as the National Health System (NHS) closely monitor compliance with these standards, they are not consistently applied worldwide [30]. Lack of resources, as well as significant constraints on facilities and costs, influence the ability to provide adequate sterilisation. These factors have not been addressed at a whole-system level, preventing the widespread adoption of reusable textiles and a harmonised practice globally, as evidenced by the results of this study.

A previous mixed-methods study on user-end perception of transition to reusable textiles was published, which identified functionality and safety, environmental concerns, cost and user comfort as the main barriers to transition. This focused on a single-centre in a HIC, and does not benefit from mapping to an implementation science framework [31]. A qualitative exploration of barriers and facilitators for the replacement of single-use device with reusable ones, conducted in a tertiary centre in Australia, was informed by a different implementation science framework. The authors suggested further research could benefit from mapping to the CFIR framework [32].

### Strengths and limitations

This study represents an important addition to the current literature as it provides a detailed and generalisable account of the perceived barriers to the introduction of reusable textiles in operating theatres globally. It includes 5230 responses from peri-operative professionals in hospitals across a broad range of countries and income groups, enhancing the generalisability of the results to diverse contexts. Additionally, the study benefits from mapping the barriers identified during qualitative analysis to the widely used CFIR framework. This approach provides insights into the organisational, institutional, and individual-level factors that can be addressed to influence the uptake of reusable drapes and gowns.

Limitations of the study include the range of the respondents, who were self-selected and did not include professionals from wider hospital management, sterilisation facilities or estate services. Inclusion of these professionals could have provided further insight into the existing barriers to implementation. Self-selection could also mean that those who responded to the survey on sustainability practices are those individuals with an interest in sustainability, which could have biased the result towards the generally positive outlook on these practices. Moreover, as the survey is based on a self-reporting questionnaire, there might discrepancies between the views provided by the respondents and the local practices. Specifically, the survey did not capture information on local textile practices utilised by the respondents, while their habitual practice represents a strong factor influencing the respondents’ views on the topic. The closed-question section of the survey aimed to assess respondents’ views on patient safety, unintended detrimental consequences and likelihood of successful implementation of the reusable drapes and gowns. A Likert scale was used to collect responses (Very likely, Likely, Neutral, Unlikely, Very Unlikely) but these were not clearly defined. Using a different method such as a discreet choice experiment could have allowed more precise interpretation of the results. Additionally, as free-text content was gathered through a survey, the responses given were often short and pragmatic, missing a reflexive element which could have provided a more nuanced understanding of the barriers. The study could have benefitted from the identification of the complementary facilitators to the barriers, which could have provided further insight including on implementation processes.

### Implication for practice and future research

This paper represents an early phase study collecting the views of the frontline teams on the use of reusable textiles and establishes baseline knowledge among clinical teams. It highlights the need for further research to inform global guidance and practice worldwide. In particular, research needs to address the clinical impact of the reusable drapes and gowns, considering the constraints reported on sterilisation processes and infrastructure, and with these the ability to guarantee adequate sterilisation. Further evidence on the cost-effectiveness of the reusable textiles across different contexts is also required, as this was widely recognised as a barrier to implementation.

A major cluster-randomised trial (Disposable versus Reusable drApes and Gowns for green OperatiNg theatres, DRAGON trial) has been planned to address these gaps in knowledge, generating high quality evidence on clinical outcomes, cost-effectiveness and carbon impact for reusable and disposable drapes and gowns that is relevant to institutions in high and lower resource settings. The trial will also involve a process evaluation, which will provide further insight into the process of implementation of reusable textiles at an individual, organisational and wider system level. The programme theory will be further refined and tested in this trial. Importantly, the process evaluation will target groups that were difficult to access with the survey in the present study due to its dissemination methods in order to expand and refine the programme theory in ways that are sensitive to context. Future respondents are expected to include procurement managers, staff from sterilisation facilities and waste managers. Through interviews and focus groups involving these professionals we aim to obtain more reflexive insights into the perceived barriers and facilitators to the use of reusable and disposable drapes and gowns, addressing the limitations of the present study. In the meantime, the strategy identification tool (Fig. 2) provides a starting point for an institution wishing to introduce reusable drapes and gowns. To optimise its use in hospitals, we recommend identifying and involving the key stakeholders to explore the tool, to help develop implementation strategies tailored to the institution needs and overcome the context-specific barriers.

## Conclusion

This paper represents a unique study filling the gap at the intersection between implementation science and environmentally sustainable surgery. With health systems striving to reach net-zero, implementation science is likely to acquire a larger role in sustainable surgery, to allow effective implementation of decarbonising practices within operating theatres and the wider hospital setting. This study identified barriers to the implementation of reusable drapes and gowns at a global scale, as perceived by a wide variety of respondents across different settings and contextualised them. Embedding the study within implementation research, by mapping of the barriers to the CFIR framework and developing a programme theory related to the intervention, makes the knowledge derived from this study transferrable, and applicable to other studies and a wide range of different sustainable interventions. Therefore, this paper a key piece of literature to guide future research and implementation strategies in environmentally sustainable surgery.

## Supplementary Information

Supplementary Material 1.

## Data Availability

The datasets used and/or analysed during the current study are available from the corresponding author (VL) on reasonable request.

## Abbreviations

COM-B: Capability Opportunity Motivation- Behaviour
CFIR: Consolidated Framework for Implementation Research
DRAGON: Disposable versus Reusable drApes and Gowns for green OperatiNg theatres trial
HICs: High-income countries
ISO: International Organization for Standardization
LICs: Low-income countries
LMICs: Lower middle-income countries
NHS: National Health System
NIHR: National Institute for Health and Care Research
UMICs: Upper middle-income countries
WHO: World Health Organisation
