# Supplementary material for: The implementation of reusable drapes and gowns in operating theatres: A mixed-methods analysis of data from 5230 peri-operative professionals in 134 countries
Source: Implement Sci Commun. 2025 Jun 2;6:70. doi: 10.1186/s43058-025-00732-x (PMC12128376; doi:10.1186/s43058-025-00732-x)
Supplement: Supplementary file 1 — Supplementary Material 1. [file 43058_2025_732_MOESM1_ESM.docx]

**The implementation of reusable drapes and gowns in operating theatres: A mixed-methods analysis of data from 5230 peri-operative professionals in 134 countries**

**Authors:** Virginia Ledda, Aneel Bhangu, James Glasbey, Elizabeth Li, Antje Lindenmeyer, Sivesh Kamarajah, Dion Mortons, Maria Picciochi, Dmitri Nepogodiev*, Laura Kudrna*

*joint senior authors

On behalf of the *National Institute for Health and Care Research Global Health Research Unit on Global Surgery*

Collaborating authors are listed in the Acknowlegdement section

**Corresponding author:**

Miss Virginia Ledda, NIHR Global Health Research Unit on Global Surgery, Institute of Applied Health Research, University of Birmingham, Birmingham B15 2TH, UK

[vxl275@student.bham.ac.uk](file:///Users/vxl275/Desktop/vxl275@student.bham.ac.uk)

**Funding:** This study is funded by the NIHR (NIHR 204403: Developing environmentally sustainable operating theatres and NIHR156087 Environmentally sustainable hospitals in Low- and Middle-Income Countries). The views expressed are those of the authors and not necessarily those of the NIHR.

| Appendix 1 – Supplementary materials | Pages 2-14 |
| --- | --- |

**Appendix 1: Supplementary materials**

| **Supplementary materials titles** | Pages |
| --- | --- |
| Supplementary Material 1: SRQR reporting checklist | 3-4 |
| Supplementary Material 2: Researcher characteristics and reflexivity | 5 |
| Table S1: Survey questionnaire | 6 |
| Figure S1: Flowchart of responses included in the mixed-methods study | 7 |
| Table S2: Responses to close-ended questions of the global survey, summarised by income group | 8 |
| Table S3: Coding frame with description of each sub-code | 9-10 |
| Table S4: Subgroup analysis of respondents who stated the intervention was ‘Unlikely’ or ‘Very Unlikely’ to be successfully implemented | 11 |
| Table S5: Frequency of coding of barriers within the orthopaedic surgeons’ subgroup | 12 |
| Figure S2: Codes and sub-codes in the coding frame mapped to constructs and subconstructs in the CFIR framework | 13 |
| Figure S3: Programme theory about the implementation of reusable drapes and gowns | 14 |

### **Supplementary material 1. SRQR reporting checklist**

| **Standards for Reporting Qualitative Research (SRQR)*** |  | |  |
| --- | --- | --- | --- |
| <http://www.equator-network.org/reporting-guidelines/srqr/> |  | |  |
|  | **Page/line no(s).** | |  |
| **Title and abstract** | |  | |
| **Title** - Concise description of the nature and topic of the study Identifying the study as qualitative or indicating the approach (e.g., ethnography, grounded theory) or data collection methods (e.g., interview, focus group) is recommended | 1 | |  |
| **Abstract** - Summary of key elements of the study using the abstract format of the intended publication; typically includes background, purpose, methods, results, and conclusions | 4 | |  |
|  |  | |  |
| **Introduction** | |  | |
| **Problem formulation** - Description and significance of the problem/phenomenon studied; review of relevant theory and empirical work; problem statement | 5-6 | |  |
| **Purpose or research question** - Purpose of the study and specific objectives or questions | Pg 6 lines 3-8 | |  |
|  |  | |  |
| **Methods** | |  | |
| **Qualitative approach and research paradigm** - Qualitative approach (e.g., ethnography, grounded theory, case study, phenomenology, narrative research) and guiding theory if appropriate; identifying the research paradigm (e.g., postpositivist, constructivist/ interpretivist) is also recommended; rationale** | Pg 7 lines 15- page 8 line 27 | |  |
| **Researcher characteristics and reflexivity** - Researchers’ characteristics that may influence the research, including personal attributes, qualifications/experience, relationship with participants, assumptions, and/or presuppositions; potential or actual interaction between researchers’ characteristics and the research questions, approach, methods, results, and/or transferability | Supplementary material 2 | |  |
| **Context** - Setting/site and salient contextual factors; rationale** | Pg 8 line 28- page 9, line 2 | |  |
| **Sampling strategy** - How and why research participants, documents, or events were selected; criteria for deciding when no further sampling was necessary (e.g., sampling saturation); rationale** |  |  |  |
| **Ethical issues pertaining to human subjects** - Documentation of approval by an appropriate ethics review board and participant consent, or explanation for lack thereof; other confidentiality and data security issues | Pg 9, lines 3-7 | |  |
| **Data collection methods** - Types of data collected; details of data collection procedures including (as appropriate) start and stop dates of data collection and analysis, iterative process, triangulation of sources/methods, and modification of procedures in response to evolving study findings; rationale** | Pg 9, lines 8-19 | |  |
| **Data collection instruments and technologies** - Description of instruments (e.g., interview guides, questionnaires) and devices (e.g., audio recorders) used for data collection; if/how the instrument(s) changed over the course of the study | Pg 9, lines 8-19 | |  |
| **Units of study** - Number and relevant characteristics of participants, documents, or events included in the study; level of participation (could be reported in results) | Results, pages 12-13 | |  |
| **Data processing** - Methods for processing data prior to and during analysis, including transcription, data entry, data management and security, verification of data integrity, data coding, and anonymization/de-identification of excerpts | Pg 9, lines 8-19 | |  |
| **Data analysis** - Process by which inferences, themes, etc., were identified and developed, including the researchers involved in data analysis; usually references a specific paradigm or approach; rationale** | Pg 9, line 20- page 10 line 14 | |  |
| **Techniques to enhance trustworthiness** - Techniques to enhance trustworthiness and credibility of data analysis (e.g., member checking, audit trail, triangulation); rationale** | Pg 10, lines 15-18 | |  |
|  |  | |  |
| **Results/findings** | |  | |
| **Synthesis and interpretation** - Main findings (e.g., interpretations, inferences, and themes); might include development of a theory or model, or integration with prior research or theory | Pg 12-14 line 28 | |  |
| **Links to empirical data** - Evidence (e.g., quotes, field notes, text excerpts, photographs) to substantiate analytic findings | Table 2 | |  |
|  |  | |  |
| **Discussion** | |  | |
| **Integration with prior work, implications, transferability, and contribution(s) to the field -** Short summary of main findings; explanation of how findings and conclusions connect to, support, elaborate on, or challenge conclusions of earlier scholarship; discussion of scope of application/generalizability; identification of unique contribution(s) to scholarship in a discipline or field | Pg 15 | |  |
| **Limitations** - Trustworthiness and limitations of findings | Pg 16 lines 11-31 | |  |
|  |  | |  |
| **Other** | |  | |
| **Conflicts of interest** - Potential sources of influence or perceived influence on study conduct and conclusions; how these were managed | Declarations, page 2 | |  |
| **Funding** - Sources of funding and other support; role of funders in data collection, interpretation, and reporting | Declarations, page 2 | |  |

**Supplementary Material 2. Researcher characteristics and reflexivity**

Virginia Ledda (VL) is a second year PhD fellow, surgical registrar in the United Kingdom and has recently started gaining experience in qualitative research methods through methods courses at the University of Birmingham.

Laura Kudrna (LK) is an Assistant Professor in Health Research Methods at the University of Birmingham and has broad experience in behaviour change and qualitative research methods.

Dmitri Nepogodiev (DN) is an Academic Clinical Lecturer at the University of Birmingham and public health registrar. He specialises in public health and surgical epidemiology.

Aneel Bhangu (AB) is a Professor of Global Surgery at the University of Birmingham and a Consultant Colorectal Surgeon at University Hospital Birmingham. He has an international research portfolio and conducts clinical trials globally.

**Table S1. Survey questionnaire**

**Figure S1. Flowchart of responses included in the mixed-methods study**

Responses for quantitative analysis

1563 responses providing free-text content

5230 responses included

Green Surgery Round 2 Survey

5734 responses

49 incomplete responses or not related to reusable drapes and gowns

Responses for qualitative

analysis

1514 free-text responses on barriers to introducing reusable drapes and gowns

Excluded:

Not eligible- 45 responses

Blank- 188 responses

Duplicates- 271 responses

3667 responses not providing free-text content

**Table S2. Responses to close-ended questions of the global survey, summarised by income group**

| How likely do you think it is that this intervention could have a negative impact on patient safety? | | | | | |
| --- | --- | --- | --- | --- | --- |
|  | **High income (n=2995)** | **UMIC (n=879)** | **LMIC (n=1069)** | **Low income (n=287)** | **Total (n=5230)** |
| Very likely | 121 (4.0%) | 77 (8.7%) | 72 (6.7%) | 37 (12.9%) | **307 (5.9%)** |
| Likely | 371 (12.4%) | 173 (19.7%) | 234 (21.9%) | 80 (27.9%) | **858 (16.4%)** |
| Neutral | 493 (16.5%) | 130 (14.8%) | 135 (12.6%) | 40 (13.9%) | **798 (15.3%)** |
| Unlikely | 1091 (36.4%) | 282 (32.1%) | 374 (35.0%) | 89 (31.0%) | **1836 (35.1%)** |
| Very unlikely | 919 (30.7%) | 217 (24.7%) | 254 (23.8%) | 40 (13.9%) | **1430 (27.3%)** |
| *Missing* | 0 (0%) | 0 (0%) | 0 (0%) | 1 (0.3%) | **1 (0.0%)** |

| How likely do you think it is that this intervention could have other unintended detrimental consequences for the environment or patient care? | | | | | |
| --- | --- | --- | --- | --- | --- |
|  | **High income (n=2995)** | **UMIC (n=879)** | **LMIC (n=1069)** | **Low income (n=287)** | **Total (n=5230)** |
| Very likely | 90 (3.0%) | 54 (6.1%) | 44 (4.1%) | 27 (9.4%) | **215 (4.1%)** |
| Likely | 473 (15.8%) | 193 (22.0%) | 272 (25.4%) | 83 (28.9%) | **1021 (19.5%)** |
| Neutral | 600 (20.0%) | 171 (19.5%) | 174 (16.3%) | 57 (19.9%) | **1002 (19.2%)** |
| Unlikely | 1086 (36.3%) | 277 (31.5%) | 350 (32.7%) | 77 (26.8%) | **1790 (34.2%)** |
| Very unlikely | 745 (24.9%) | 184 (20.9%) | 229 (21.4%) | 42 (14.6%) | **1200 (22.9%)** |
| *Missing* | 1 (0.0%) | 0 (0%) | 0 (0%) | 1 (0.3%) | **2 (0.0%)** |

| How likely do you think it is that this intervention could be successfully implemented in your hospital? | | | | | |
| --- | --- | --- | --- | --- | --- |
|  | **High income (n=2995)** | **UMIC (n=879)** | **LMIC (n=1069)** | **Low income (n=287)** | **Total (n=5230)** |
| Very likely | 461 (15.4%) | 213 (24.2%) | 385 (36.0%) | 103 (36.0%) | **1162 (22.2%)** |
| Likely | 930 (31.0%) | 320 (36.4%) | 384 (35.9%) | 92 (32.0%) | **1726 (33.0%)** |
| Neutral | 637 (21.3%) | 147 (16.7%) | 136 (12.7%) | 38 (13.2%) | **958 (18.3%)** |
| Unlikely | 690 (23.0%) | 133 (15.1%) | 122 (11.4%) | 39 (13.6%) | **984 (18.8)** |
| Very unlikely | 277 (9.3%) | 66 (7.5%) | 42 (3.9%) | 14 (4.9%) | **399 (7.6%)** |
| *Missing* | 0 (0%) | 0 (0%) | 0 (0%) | 1 (0.3%) | **1 (0.0%)** |

**UMIC: Upper middle-income countries, LMIC: lower middle-income countries.**

**Table S3. Coding frame with description of each sub-code**

| **Codes** | **Sub-codes** | **Description** | | | |
| --- | --- | --- | --- | --- | --- |
| **Evidence** | **Further evidence required** | Lack of evidence proving the benefits of the intervention described as a barrier to implementation | | | |
|  | **Lack of evidence- Asepsis** | Lack of evidence proving that the intervention is equal or more beneficial compared to current practice with regards to asepsis and infection control | | | |
|  | **Lack of evidence- Cost benefit** | Lack of evidence proving that the intervention is equal or more beneficial compared to current practice with regards to cost | | | |
|  | **Lack of evidence- Environmental benefit** | Lack of evidence proving that the intervention is equal or more beneficial compared to current practice with regards to environmental impact | | | |
| **Finance** | **Cost** | Cost of the intervention is described as a barrier to implementation | | | |
|  | **Cost of initial investment** | Cost of the initial investment required to introduce intervention | | | |
|  | **Cost of sterilisation** | Cost of sterilisation of drapes and gowns | | | |
|  | **Cost of workforce** | Cost of the workforce required to implement intervention | | | |
|  | **Cost-other** | Other financial barriers including lack of funding to the hospital, need to charge higher prices with disposable | | | |
| **Institution** | **Institutional barriers** | Responses which are related to the management of an institution (hospital) | | | |
|  | **Bureaucratic barriers** | Reponses which identify complicated or lengthy administrative procedure or processes as the main barrier | | | |
|  | **Environmental sustainability not prioritised** | Responses reporting that environmental sustainability and interventions aiming to improve this are not prioritised within the institution | | | |
|  | **Resistance from stakeholders** | Responses describing resistance to change towards the intervention from stakeholders including peri-operative professionals and patients | | | |
|  | **Institutional guideline or policy** | Responses which indicate existing policy or guidelines within an institution (hospital) the lack of guidelines and policies promoting the intervention as a barrier for the intervention | | | |
|  |  | **Approval from Infection Control team** | Infection control policies opposing the introduction of reusable textiles or infection control teams having to approve intervention | | |
|  | **Previous switch to disposable** | Responses relating to institutions that previously used reusable and then moved on to disposable textiles: subsequent changes in workflow and facilities make switching back to reusable textiles challenging | | | |
|  | **Change in workflow** | Responses indicating that the intervention would require a change in processes, logistics and procedures within an institution, which represents a barrier to the implementation | | | |
|  | **Lack of motivation** | Lack of motivation or commitment from staff within an institution to implement intervention | | | |
|  | **Single- use culture** | Responses indicating use of disposable items as part of a habit within an institution | | | |
| **Intervention** | **Quality of drapes and gowns** | Responses indicating material features of the drapes and gowns and their durability including tearing during sterilisation and need for replacements as a result | | | |
|  | **Practicality and Comfort** | Responses indicating practicality, ease of use and comfort as main reasons why disposable drapes and gowns are preferred to reusables. | | | |
|  | **Negative perception of intervention** | Reponses indicating negative perceptions of reusable drapes and gowns as the barrier. These can include the intervention being perceived as inadequate, retrograde. | | | |
|  | **Permeability of reusables** | Reusables being permeable to liquids (or disposable being waterproof) indicated as the barrier to implementation | | | |
| **Procurement** | **Procurement** | The process of sourcing, ordering, procuring, or obtaining the reusable drapes and gowns indicated as a barrier to implementation, including lack of contracts with reusable textiles suppliers, or existing contracts with disposable textile suppliers | | | |
|  | **Shortage in supply of reusables** | Low supply or poor availability of reusable textiles indicated as barrier | | | |
|  |  | **Sterilisation causing delay in supply** | | Sterilisation process leading to a delay in supply due to low capacity | |
| **Resources** | **Additional resources required** | Lack of resources in local setting needed to implement intervention successfully or additional resources required compared to existing in order to effectively implement the intervention | | | |
|  | **Additional workforce required** | Lack of workforce or additional workforce required (compared to existing) to implement the intervention successfully | | | |
|  | **Additional workload with reusables** | Implementation of the intervention would lead to increase in workload for hospital workers/ sterilisation personnel which is perceived as a barrier | | | |
|  | **Additional time required** | Additional time required to ensure intervention is effectively implemented within the institution | | | |
| **Safety** | **Concerns about safety** | Responses describing concerns about breaching safety of patients and staff by using reusable drapes and gowns | | | |
|  | **Concerns about infection/ contamination** | Responses expressing concerns about increased risk of infection transmission, contamination of patient and staff when using reusable drapes and gowns | | | |
|  | **Disposable to be used for high-risk cases** | Responses indicating that disposable textiles are still being used in high-risk cases including operations on known infected patients (COVID-19, HIV, Hepatitis) or clean procedure with use of surgical implants | | | |
| **Sterilisation** | **Sterilisation process** | Responses indicating the need to introduce a sterilisation system or process, or increase sterilisation capacity | | | |
|  |  | **Lack of basic utilities** | | | Lack of utilities such as water and electricity leading to reliable sterilisation not being available |
|  | **Sterilisation equipment** | Sterilisation equipment or facilities intended as physical spaces and infrastructures recognised as barrier for implementation of the intervention. This includes sterilisation equipment which is faulty or prone to breakdown or physical barriers that do not allow appropriate sterilisation | | | |
|  | **Concerns about inadequate sterilisation** | Responses expressing concerns about sterilisation not being adequate or compliant to standards, with potential to lead to increased risk of infection transmission and contamination | | | |
|  | **Sterilisation-other** | Other issues pertaining to sterilisation including need for sterilisation standards and monitoring of sterilisation quality | | | |
| **Other** | **Education and training** | Education and training of:   - peri-operative staff with regards to the benefits of the intervention (cost- benefit, environmental benefit, and infection control). - Sterilisation service staff with regards to sterilisation process and monitoring. | | | |
|  | **Environmental concern** | Responses expressing concerns that the environmental impact of the intervention could be increased with reusable textiles compared to disposable. | | | |
|  | **External barriers** | Responses indicating companies or organisations external to the institution posing limitation to adopting the intervention by regulations or authorisations | | | |
|  | **Individual preference** | Individuals choosing to use disposable drapes and gowns as their use appeals to them more than the reusable textiles or who are used to using disposable textiles, making change to reusable challenging | | | |
|  | **Other** | Any other reasons listed by respondents that were not appliable to any of the above domains | | | |

**Table S4. Subgroup analysis of respondents who stated the intervention was ‘Unlikely’ or ‘Very Unlikely’ to be successfully implemented**

|  | Evidence | Finance | Institution | Intervention | Other | Procurement | Resources | Safety | Sterilisation |
| --- | --- | --- | --- | --- | --- | --- | --- | --- | --- |
| HIC  (n=389) | 12 (3.1%) | 87 (22.4%) | 83 (21.3%) | 16 (4.1%) | 52(13.4%) | 20 (5.1%) | 21 (5.4%) | 44(11.3%) | 54 (13.9%) |
| UMIC  (n=47) | 2 (4.3%) | 13 (27.7%) | 9 (19.1%) | 2 (4.3%) | 3 (6.4%) | 4 (8.5%) | 1 (2.1%) | 4 (8.5%) | 9 (19.1%) |
| LMIC  (n=43) | 3 (7.0%) | 10 (23.2%) | 3 (7.0%) | 0 (0.0%) | 4 (9.3%) | 2 (4.7%) | 4 (9.3%) | 4 (9.3%) | 13 (30.2%) |
| LIC  (n=10) | 0 (0.0%) | 7 (70%) | 1 (10.0%) | 0 (0.0%) | 0 (0.0%) | 0 (0.0%) | 1 (10.0%) | 0 (0.0%) | 1 (10.0%) |
| Total  (n=488) | **17 (3.5%)** | **117 (24.0%)** | **97 (19.9%)** | **18 (3.7%)** | **58 (11.9%)** | **26 (5.3%)** | **27 (5.5%)** | **51 (10.4%)** | **77 (15.8%)** |

**HIC: high-income countries, UMIC: Upper middle-income countries, LMIC: lower middle-income countries, LIC: low-income countries.**

**Table S5: Frequency of coding of barriers within the orthopaedic surgeons’ subgroup**

|  | Frequency of coding |
| --- | --- |
| Evidence | 8 (6.1%) |
| Financial | 26 (19.8%) |
| Institution | 12 (9.2%) |
| Intervention | 16 (12.2%) |
| Procurement | 13 (9.9%) |
| Resources | 4 (3.1%) |
| Safety | 17 (13.0%) |
| Sterilisation | 20 (15.3%) |
| Other | 15 (11.4%) |
| Total | **131** |

**Figure S2. Codes and sub-codes in the coding frame mapped to constructs and subconstructs in the CFIR framework**

**
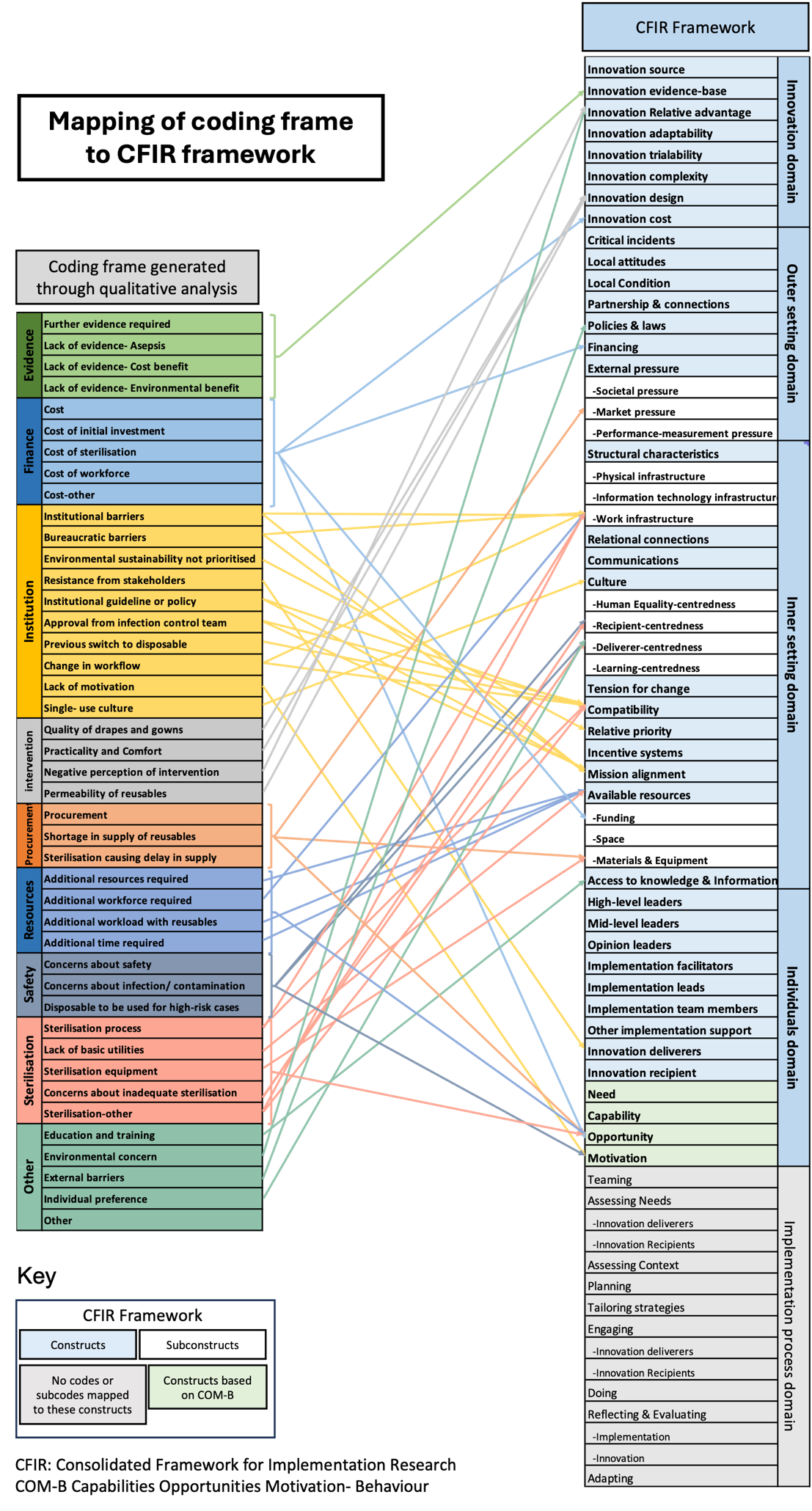
**

**Figure S3. Programme theory about the implementation of reusable drapes and gowns**

**
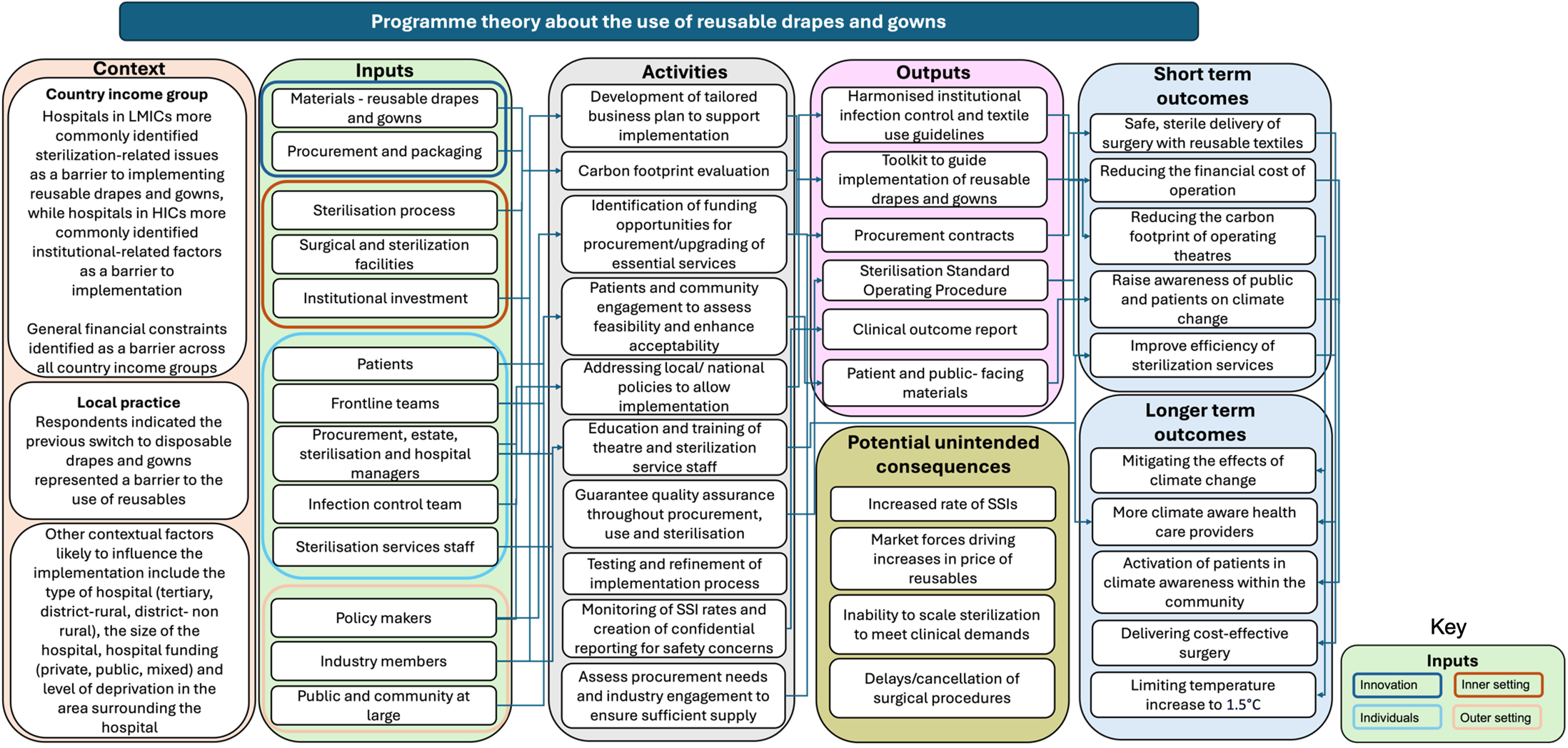
**

**LMICs: low- and middle-income countries, HICs: High income countries, SSIs: Surgical site infections.**
